# Transforming Anxieties of Aging in South-Eastern Europe: A Public Health Humanities Initiative

**DOI:** 10.1093/geroni/igaf122.3254

**Published:** 2025-12-31

**Authors:** Ulla Kriebernegg

**Affiliations:** University of Graz, Graz, Steiermark, Austria

## Abstract

This paper presents the interdisciplinary research project “Transforming Anxieties of Aging in South-Eastern Europe (2023–2027)”, funded by the German Volkswagen Foundation. The project investigates demographic nationalism in the region, where rapid population aging is increasingly framed as a societal “burden.” South-Eastern Europe has transitioned from high population growth to the fastest aging rates in Europe, with alarmist narratives in media and policy shaping attitudes toward aging and migration. The COVID-19 pandemic further exacerbated these issues, with the region experiencing some of the highest global mortality rates, particularly among older populations. Using an innovative Public Health Humanities approach, this project explores how ageist narratives articulate broader societal anxieties—ranging from concerns over the welfare state and territorial disputes to migration and shifting minority demographics. By examining how fears are transferred across different social domains, the study critically analyzes the metaphors and framings of demographic change. A transnational, interdisciplinary team from nine countries integrates Age Studies and Area Studies, combining qualitative and quantitative methods from social history, anthropology, sociology, demography, political science, cultural studies, and literary studies. The project employs an intersectional, participatory Citizen Science approach to reshape aging narratives—moving beyond alarmist portrayals toward empowering perspectives. Reflexively addressing “age bias” within the research team itself, this study highlights the essential role of Age Studies / Cultural Gerontology in understanding demographic discourse and developing socially just, intergenerationally inclusive policies. The paper will argue that the project can be framed as a Public Health Humanities Initiative that opens new horizons in (Cultural) Gerontology.

